# Acute Myocardial Infarction during the COVID-19 Pandemic: Long-Term Outcomes and Prognosis—A Systematic Review

**DOI:** 10.3390/life14020202

**Published:** 2024-01-31

**Authors:** Marius Rus, Adriana Ioana Ardelean, Felicia Liana Andronie-Cioara, Georgiana Carmen Filimon

**Affiliations:** 1 Department of Medical Disciplines, Faculty of Medicine and Pharmacy, University of Oradea, 410073 Oradea, Romania; 2 Department of Preclinical Disciplines, Faculty of Medicine and Pharmacy, University of Oradea, 410073 Oradea, Romania; adriana_toadere@yahoo.com (A.I.A.); georgiana.cartis@gmail.com (G.C.F.); 3 Department of Psycho Neuroscience and Recovery, Faculty of Medicine and Pharmacy, University of Oradea, 410073 Oradea, Romania

**Keywords:** COVID-19, myocardial infarction, cardiovascular burden, acute coronary syndromes

## Abstract

Coronavirus disease 2019 (COVID-19) was a global pandemic with high mortality and morbidity that led to an increased health burden all over the world. Although the virus mostly affects the pulmonary tract, cardiovascular implications are often observed among COVID-19 patients and are predictive of poor outcomes. Increased values of myocardial biomarkers such as troponin I or NT-proBNP were proven to be risk factors for respiratory failure. Although the risk of acute coronary syndromes (ACSs) was greater in the acute phase of COVID-19, there were lower rates of hospitalization for ACSs, due to patients’ hesitation in presenting at the hospital. Hospitalized ACSs patients with COVID-19 infection had a prolonged symptom-to-first-medical-contact time, and longer door-to-balloon time. The mechanisms of myocardial injury in COVID-19 patients are still not entirely clear; however, the most frequently implicated factors include the downregulation of ACE2 receptors, endothelial dysfunction, pro-coagulant status, and increased levels of pro-inflammatory cytokines. The aim of this paper is to evaluate the long-term outcomes and prognosis of COVID-19 survivors that presented an acute myocardial infarction, by reviewing existing data. The importance of the association between this infectious disease and myocardial infarction arises from the increased mortality of patients with SARS-CoV-2 infection and AMI (10–76%, compared with 4.6% for NSTEMI patients and 7% for STEMI patients without COVID-19). The literature review showed an increased risk of cardiovascular events in COVID-19 survivors compared with the general population, even after the acute phase of the disease, with poorer long-term outcomes.

## 1. Introduction

By June 2023, more than seven hundred million people were infected with coronavirus, and more than six million deaths had been registered worldwide [1]. 

Although the virus primarily affects the respiratory tract, causing a mild-to-severe case of pneumonia and ultimately severe acute respiratory syndrome (SARS), myocardial injury is frequently observed in patients with COVID-19 (defined as increased levels of cardiac necrosis biomarkers, especially high-sensitivity troponin). One of the implicated mechanisms is the fact that angiotensin-converting enzyme 2 (ACE2) works as a receptor for coronavirus, and that this enzyme is highly expressed in the heart and lungs [2].

Studies have shown that an increase in troponin levels among COVID-19 patients was associated with higher mortality [3]. Myocardial injury can have different electrocardiographic expressions, from a normal EKG to an ST-elevated acute myocardial infarction. 

In this review, we studied the incidence, possible mechanisms, and specific challenges of acute myocardial infarction in patients with COVID-19.

Previous studies reported that viral infections involving the respiratory tract are potential risk factors for acute coronary syndromes (ACSs). One of the possible mechanisms of type 1 AMI (acute myocardial infarction) is the pro-inflammatory state, which can promote the destabilization of a coronary atherosclerotic plaque [4]. Type 2 AMI, also known as myocardial infarction with non-obstructive coronary arteries (MINOCA), could be promoted by hypoxia, tachycardia, and hypotension—symptoms that appear in acute respiratory failure [5]. 

In SARS-CoV-2 infection, there are particular pathways that can induce AMI, such as endothelial and microvascular injuries, coronary vasospasm, thrombosis, increased platelet consumption, and the cytokine storm [6]. However, there is little information about the post-SARS-CoV-2 infection sequelae. Due to the multiple pathophysiological mechanisms involved in the occurrence of ischemic coronary disease that appear as a result of SARS-CoV-2 infection, we considered it important to evaluate the implications of the coexistence of these two entities.

The present manuscript aims to evaluate the outcomes and long-term prognosis of patients with acute coronary syndrome and post-SARS-CoV-2 infection status by performing a systematic review of the available data. 

## 2. Method

The study followed the Preferred Reporting Items for Systematic Reviews and Meta-Analyses (PRISMA) reporting guidelines, reported in Supplementary Table S1. The authors used the PubMed platform and Google Scholar to search articles published from February 2020 to 2023, using the keywords “COVID” and “myocardial infarction”. Review articles, duplicate articles, opinion letters, animal studies, corrections, and articles not relevant to cardiology were excluded. The search was limited to articles published in English. Research reviews and record screenings were carried out by two authors (G.C.F. and A.I.A.) separately to extract data. Any differences in opinion, questions, and discordant data were resolved by consulting a third author (M.R.). The inclusion criteria for research studies were (1) studies involving the enrollment of patients with COVID-19 infection and acute coronary syndromes; (2) studies with follow-up ≥30 days; and (3) studies investigating primary cardiovascular outcomes. The exclusion criteria were (1) small-cohort studies including fewer than 100 patients; (2) studies with short-term follow-up data; (3) studies with no conclusive data for AMI incidence; and (4) studies with no control group. 

## 3. Results

**Figure 1:**
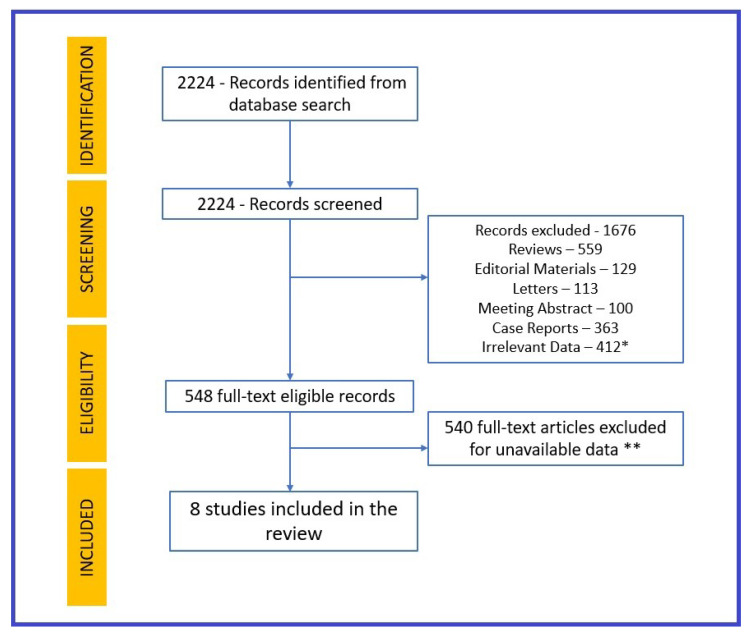
Research process. * Articles excluded because of no data about in-hospital AMI incidence and mortality. ** Articles excluded because they presented no data about long-term outcomes of AMI patients with COVID-19.

There were 2224 results that fit our initial search, and after screening through our exclusion criteria, 548 articles remained. After full-text reading, 540 articles were excluded, leaving 8 studies relevant to our chosen subject, which were included in our research. Each study defined AMI according to the Forth Universal Definition of Myocardial Infarction [7]. The research, screening, and review process is described in Figure 1. 

Together, the eight studies have enrolled 8,736,943 patients, of which 74% were males, including 845,258 COVID-19-positive patients, with different degrees of severity of pulmonary disease. The characteristics of the selected studies are presented in Table 1. The mean follow-up period was 21 months, ranging between 1 and 60 months. The occurrence of AMI was screened by searching in the patient’s medical data using the International Classification of Diseases 10th Revision (ICD-10) codes I21 and I22. 

The North American COVID-19 Myocardial Infarction Registry (NACMI) [8] enrolled patients from January 2020 to December 2020 with ST-elevation myocardial infarction (STEMI). The study had as a primary end-point in-hospital death, stroke, recurrent myocardial infarction and unplanned revascularization. For the accurate comparison of reperfusion strategies, patients referring at a hospital within 60 miles of a primary PCI hospital were excluded. As a result, the authors noted a slightly longer door-to-balloon time in COVID-19 patients than in the control group. The primary endpoint was met in 36% of COVID-19-positive patients and 5% of COVID-19-negative patients. This difference resulted from an increased in-hospital mortality in the first group. The mortality was higher for the patients who did not undergo coronary revascularization, and also, the patients who stayed longer in intensive care units. 

Xie et al. [9] aimed to assess the risk and one-year burdens of cardiovascular outcomes in patients recovering from COVID-19 pneumonia. The composite outcome of the study was the assessment of the burden of cardiovascular diseases, such as: stroke, dysrhythmias, ischemic heart disease, heart failure, cardiogenic shock, inflammatory heart disease, thrombotic disorders and major acute cardiac events. The authors observed a high risk of cardiovascular disease, arrhythmias, stroke, and thrombotic events at 12-month follow-up in patients who recovered from COVID-19 disease, even for the ones who were not hospitalized. The risk and associated cardiovascular burdens increased proportionally with the severity of COVID-19 pneumonia. The study also showed an increased risk of cardiovascular disease even beyond the acute phase of the infection. 

A smaller study led by Wei et al. [10] showed that patients with COVID-19 and acute myocardial injury were more likely to require admission into an intensive care unit (ICU), there was a greater need of mechanical ventilation and vasoactive agents, and death incidence was higher. The authors recognized as predictors of severe outcome older age, arterial hypertension, cerebrovascular disease, use of calcium canals blockers, lower glomerular filtration rate and elevated Troponin or C reacted protein (CRP).

Wang et al. [11] analyzed the cardiovascular risk that COVID-19 survivors showed at the one-year follow-up. For that reason, the composite outcome for cardiovascular diseases was defined as the first incidence of any complication. The followed outcomes were: stroke, arrhythmia, pericarditis, myocarditis, acute coronary disease, acute myocardial infarction, angina, heart failure, cardiac arrest, cardiogenic shock and thrombotic disease. The results of this study reveal that COVID-19 survivors have an increased mortality rate in relation to all the cardiovascular diseases. The impact of this disease on cardiovascular-related outcomes appeared to be more pronounced in hospitalized patients. The 12-month risk of incidental cardiovascular disease was higher in COVID-19-surviving patients than in the control group, and they showed a higher risk of complications. Female survivors of COVID-19 pneumonia seemed to have more frequent arrhythmias than the control group. Myocarditis and ischemic heart diseases were two of the most frequently encountered diseases in younger survivors. 

Kiris T et al. studied outcomes of patients with acute coronary syndrome in the pre-COVID-19 era, and compared them with STEMI patients in the COVID-19 era. Although the two groups were similar when it came to previous statin or antiplatelet treatment, history of diabetes mellitus, hypertension, tobacco use and previous coronary artery disease, it was observed that COVID-19-era patients had more frequent coronary thrombosis and a higher need of glycoproteins IIb/IIIa inhibitors treatment. There were no differences in obtaining post-procedural grade 3 TIMI flow in the two groups, but COVID-19 patients showed higher mortality and stent thrombosis [12]. The Turkish study observed that the STEMI patients from the COVID-19 era were younger, had lower LVEF, and treatment with glycoprotein IIb/IIIa inhibitors was needed more frequently. The presence of COVID-19 disease was an independent predictor for MACE incidence in STEMI patients observed in long-term follow-up, due mostly to a higher rate of hospitalization for heart failure. 

Another Turkish study, led by Cinar T, which analyzed one-year mortality in COVID-19 patients with acute myocardial infarction, observed that COVID-19 patients presented with higher Killip class, and more frequently needed invasive mechanical ventilation and treatment with inotropic agents [13]. At the one-year follow-up, mortality was significantly higher in the COVID-19 group (21%); one out of five patients with AMI died prior to follow-up. A Killip class greater that II and COVID-19 infection were considered independent predictors for negative outcome in short-term mortality. The COVID-19 group also presented with higher troponin levels, and associated higher Killip class. This leads to the conclusion that these patients had greater myocardial damage [14]. There is no clear conclusion regarding the mechanism of this phenomenon, and it is still unknown if the damage is based on viral myocarditis, plaque rupture due to viral inflammatory process, or type 1 AMI [15].

**Table 1:** General analysis of the reviewed patients.

Authors	1. Garcia S. et al. [8]	2. Wei JF. et al. [10]	3. Xie Y. et al. [9]	4. Wang W. et al. [11]	5. Kiris T et al. [12]	6. Cinar T et al. [13]	7. Choudry F.A. et al. [16]	8. Puha K. et al. [17]
Country	USA	China	USA	USA	Turkey	Turkey	UK	Singapore
Type of study	Prospective, multicenter, observational	Prospective, observational	Retrospective, observational	Retrospective, observational	Retrospective, multicenter, observational	Prospective, observational	Retrospective, observational	Retrospective, observational
Patients (n)	1191	101	5,791,407	2,940,988	1,748	721	466	321
Type of AMI	STEMI	NA	NA	NA	STEMI	STEMI/NSTEMI	STEMI	STEMI
Age	18–85	49	62.5	43	18–90	av. 64.7	av. 60.2	av. 59
Male	842 (70.69%)	54 (53.5%)	5,228,431 (90%)	1,241,483 (42.2%)	1325 (75.80%)	397 (55.06%)	381 (81.75%)	266 (82.86%)
COVID-19 confirmed	230	101	153,760	690,892	62	112	101	NR
Diabetes mellitus	386 (32%)	14 (13.9%)	1,321,907 (22.82%)	188,488 (6.4%)	509 (29%)	210 (29%)	158 (33.9%)	135 (42%)
Cardiac arrest	134 (11.25%)	NR	NR	NR	114 (6.5%)	NR	29 (6.2%)	19 (5.9%)
Cardiogenic shock	147 (12.3%)	NR	NR	NR	153 (8.7%)	NR	64 (13.7%)	30 (9.3%)
Smoking	384 (32.25%)	8 (7.9%)	2,560,147 (44.20%)	230,499 (7%)	554 (31.6%)	307 (42.5%)	248 (53.2%)	113 (41.4%)
History of CAD	322 (27%)	5 (5%)	NR	NR	218 (12.5%)	150 (21.4%)	131 (28.1%)	101 (31.4%)
Revascularization treatment	1101 (92.44%)	NR	NA	NA	1743 (99%)	100%	100%	100%
Hypertension	832 (69.85%)	21 (21%)	1,525,944 (26.34%)	440,998 (14.9%)	692 (39.5%)	356 (49%)	217 (46.5%)	189 (58.9%)
Primary end-point	In-hospital death, stroke, recurrent myocardial infarction, unplanned revascularization	Admission to an intensive care unit, need for mechanical ventilation, vasoactive treatment or death101	Incidence of cerebrovascular disease, dysrhythmias, ischemic heart disease, heart failure, pericarditis, myocarditis, cardiogenic shock, thrombotic disorders, MACE	Incidence of stroke, arrhythmia, pericarditis, myocarditis, ischemic coronary disease, heart failure, thrombotic disease, MACE, cardiac arrest, cardiogenic shock	MACE (all-cause mortality, heart failure, miocardialreinfarctization, cerebrovascular disease)	One-year mortality	One-year mortality	One-year cardiac-related mortality
Follow-up period	5 years	30 days	1 year	1 year	542 days	1 year	1 year	1 year

## 4. Long-Term Risk of Myocardial Infarction

In the studied population, we observe that the majority of patients were males, but even so, females more frequently developed arrhythmias, such as atrial fibrillation, atrial flutter and sinus tachycardia [9,10,11]. 

The NACMI only studied STEMI patients, and one of the main observations was that in the COVID-19 group, only 20% of the patients required coronary stent implantation, while the rest did not present any culprit lesion at the coronarography. In the COVID-19-negative group with STEMI, 93% needed stent implantation [8]. The in-hospital cardiovascular death was twice as high among COVID-19 patients, with a negative short-term prognosis; one out of three patients who survived the acute event were deceased at the 30-day follow-up. More than one-third of COVID-19 patients with STEMI presented recurrent MI within 30 days (Table 2). More than 30% of COVID-19 patients met the primary endpoint, compared to 5% of non-COVID-19 patients [8]. 

Wei et al. [10] observed that increased Troponin I was a negative prognostic factor for COVID-19 patients, leading more often to the need for mechanical ventilation, the use of vasoactive agents and longer admission to ICU (intensive care units). 

Patients with COVID-19 disease showed an increased risk of developing acute myocardial infarction within 30 days from the primary infection (HR = 2.32, burden 2.91/1000 patients), regardless of the severity of the respiratory disease. It was detected that the incidence of MI increased with the severity of the viral pneumonia. The pathological mechanism is still unclear; several studies identified micro-thrombosis of small coronary arteries as part of the pro-coagulability status. Except for myocardial infarction, other forms of ischemic heart disease, such as acute coronary disease (ACD), ischemic cardiomyopathy and angina, were more frequent than in the pre-COVID period and in the control groups. The incidence of this diseases is twice as high in COVID-19 survivors. 

The long-term burden of MI in COVID-19 survivors remains high even at 12 months after the resolution of the infective disease; this group of patients showed almost twice the risk of developing MI within a year (HR = 1.71, burden = 7.59/1000 patients).

The risk factors for acute myocardial infarction after COVID-19 disease are: older age, diabetes mellitus, male gender and follow-up length [12]. 

## 5. Long-Term Outcomes of Post-COVID-19-Positive Patients

Atrial fibrillation was found to be twice as frequent in post-COVID-19 patients. Although the incidence of other arrhythmias increased as well, AF was the most common one [11]. Female COVID-19 survivors and younger patients have shown greater risks of developing myocarditis and arrhythmias than male survivors. Older age survivors have shown an increased risk of developing pulmonary embolism and ischemic heart disease (HR = 1.87) (Table 3). 

Xie et al. brought evidence that patients infected with COVID-19 show an increased risk of cardiovascular disease, even after the first 30 days of infection. The risks include cardiovascular diseases, including cerebrovascular disorders, dysrhythmias, myocarditis, ischemic heart disease, heart failure and thromboembolic disease [10].

A significant increase in the incidence of myocarditis was observed among non-hospitalized patients at 12-month follow-up, according to Wang et al. This could have been caused by the absence of proper antiviral and anti-inflammatory treatment [11]. 

The NACVI study revealed that more than half of COVID-19 patients with STEMI developed different stages of heart failure. Also, COVID-19 patients presented an increased incidence of cardiac arrest and cardiogenic shock. 

Patients with COVID-19 pneumonia develop twice as frequent major acute cardiovascular events (MACE), even at the 12-month follow-up (HR = 1.87), with an HR of 1.64 in cardiovascular death. In the NACVI registry, 36% of STEMI patients with COVID-19 presented a further acute cardiovascular event. 

## 6. Discussion

In-hospital mortality reaches 7% for patients presenting with STEMI and 4.9% for patients with NSTEMI [18]. According to WHO, the percentage of COVID-19 pneumonia mortality in the general population is approximately 3.4%, compared with the mortality of seasonal Influenza, which is less than 1% [1]. Among hospitalized patients with COVID-19 disease, the mortality has been reported to be approximately 21% [19]. The estimated mortality within patients with COVID-19 and AMI was between 10% and 76.6% [20,21]. 

For a long period of time, the risk of ACSs during or after an acute infection was studied, due to the major impact of pro-inflammatory factors in the atherosclerotic process, and because of the increased number of patients presenting myocardial injury [22]. Studies carried out during the COVID-19 pandemic showed that the risk of AMI, although high in the acute period, returns to baseline after several months, and that patients with severe pneumonia were more likely to develop an acute coronary syndrome [23]. 

The potential causes of cardiovascular disease in COVID-19 infection include: the damage from direct viral invasion of cardiomyocytes, causing cell death, endothelial cell infection, the transcriptional alteration of multiple cell types, complement activation and complement-mediated coagulopathy and microangiopathy, the downregulation of ACE2 receptors and the dysregulation of the renin–angiotensin–aldosterone system, autonomic dysfunction, and elevated levels of pro-inflammatory cytokines that can induce fibrosis and scarring of cardiac tissue [24,25,26]. When the SARS-CoV-2 virus binds with ACE2 receptors, it leads to the downregulation of these receptors, and this increases the activity of angiotensin II. This mechanism results in systemic vasoconstriction, apoptosis, inflammation, and endothelial proliferation, leading to cardiomyocyte damage or the worsening of previous ischemic condition [27]. Another identified mechanism of acute myocardial infarction in COVID-19 patients is coronary embolism, found in 3% of AMI patients [28]. 

A newer hypothesis claims that the integration of the SARS-CoV-2 genome into human DNA might express as chimeric transcripts fusing viral with cellular sequences [29]. The latest data sustain the hypothesis that coronary artery thrombosis is more frequent in COVID-19 patients because of the disruption of coronary atherosclerotic pre-existing plaque, due to an increased inflammatory status and pro-inflammatory blood cells. Dilated and hypertrophied cardiomyocytes express higher amounts of the ACE2 receptors, which could explain the clinical deterioration of patients with previous cardiac diseases infected with COVID-19 [30]. Studies indicate that the association between STEMI and COVID-19 infection leads to an increased incidence of stent thrombosis, as a result of elevated thrombus burden [31]. 

There are several receptors that have been incriminated in SARS-CoV-2 infection, and ACE2 appears to be the most important. However, there are other receptors that interact with the S protein of SARS-CoV-2 virus, which may be implicated in the long-term damage caused by the virus, such as: CD147, neuropilin-1, dipeptidyl peptidase 4, alanyl aminopeptidase, and glutamyl aminopeptidase [32]. Avolio et al. conducted an experimental study that clearly showed that the S protein binds with the CD147 in human cardiac pericytes [33]. This study determined the presence of the SARS-CoV-2 S protein in the peripheral blood of COVID-19 patients, even after the infection was cured, and that S protein alone can induce the damage to the endothelial coronary arteries without infecting the cells. The effects of the S protein in the myocardial pericytes are: increased cardiac pericyte migration, reduced endothelial cell network formation, stimulated pericyte cytokine secretion, and increased levels of pro-apoptotic factors, leading to endothelial cell death [34]. These data suggest that the S protein could contribute to the long-term health impairment of COVID-19-surviving patients [35]. There are earlier studies that showed that SARS-CoV-2 uses the endosomal cysteine proteases cathepsins B/L (CTSL and/or CTSB) for cell entry, and that atrial and ventricular cardiomyocytes are possibly susceptible to SARS-CoV-2 infection by involving the CTSB/CTSL for S protein priming [36].

The involvement of CD147 in the non-infectious damage of SARS-CoV-2 in human organs has therapeutical implications, leading to the development of a humanized anti-CD147 antibody, such as meplazumab. The treatment with meplazumab in COVID-19 patients improved their condition and the recovery rate of patients, with a good safety profile [37]. Statins also decrease the actions of the S protein on the CD147 receptor [38].

Analyzing data from the reviewed studies, we observed that COVID-19 survivors presented an increased 30-day risk of stroke, increased risk of thrombotic events and inflammatory cardiac disease (mostly myocarditis), and higher risk of developing dysrhythmias [8,9,10,11]. Patients that required hospitalization for COVID-19 pneumonia were more likely to present cardiovascular events such as heart failure, acute coronary syndromes, atrial fibrillation and stroke. The risk becomes higher for patients admitted into the ICU. 

Survivors of COVID-19 have shown an increased risk and burdens of cardiovascular disease at 12-month follow-up. This increased risk was observed even in patients without a history of cardiovascular disease and with low cardiovascular risk, proving that COVID-19 disease has a direct involvement in the worsening of the prognosis. The possible pathophysiological mechanisms involved in the increased incidence of cardiovascular diseases at one year of follow-up in patients who have suffered an infection with COVID-19 are: prolonged inflammation, cellular direct damage of viral infection, cardiac myositis, cardiac fibrosis, and the binding of the S protein with myocardial pericytes [39].

Patients with COVID-19 disease who did not require hospitalization presented a higher risk of cardiovascular events, compared to the general population. There was a significant increase in cardiovascular events concurrently with the severity of the viral disease [9]. The most frequent cardiac complications were: heart failure, dysrhythmias, pericarditis, myocarditis, and ischemic heart disease.

Diabetes mellitus remains a major risk factor for myocardial infarction, both in the pre-COVID-19 period and during the COVID-19 pandemic; 46% of the patients that referred to the hospital with STEMI and COVID-19 disease had a history of diabetes mellitus, and they had a higher risk of cardiogenic shock [8]. The association between COVID-19 disease and STEMI confers a negative prognosis, resulting in the deaths of one out of three patients, according to the NACMI registry. In total, 20% of COVID-19 patients did not have a culprit vessel, and therefore did not need PCI, upholding the direct injury of myocytes as a possible mechanism for ACSs in this viral infection [8]. 

The fact that cardiovascular risk expands even at 12 months after the acute phase of COVID-19 imposes the need for a better primary prevention of COVID-19 infection, a thorough screening for cardiovascular disease and a follow-up for post-COVID-19 patients. 

Earlier studies have shown that COVID-19 infection could lead to irreversible damage to cardiovascular and respiratory systems, such as congestive heart disease or chronic cell hypoxia, increasing the risk of arrhythmias, cardiogenic shock, ischemic heart disease, and ischemic stroke [40]. 

Regarding the comparison of the risk for cardiovascular disease after COVID-19 and other infective diseases involving the respiratory tract, Daugherty et al. claimed that there is a 1.65% higher risk of a cardiovascular sequelae appearing in the post-acute phase of COVID-19 than in other viral diseases [41]. Post-acute sequelae of COVID-19 are defined as signs or symptoms that persist beyond 30 days after the infection. In the large study conducted by Daughtery (over 9 million patients), 14% of COVID-19 survivors developed a clinical sequela after the acute phase of the disease—4% more frequently than in the general population. The risk increased with age, pre-existing conditions and severity of COVID-19 pneumonia, although younger adults with no comorbidities who have SARS-CoV-2 infection also have a higher risk of developing a clinical sequela. Cohen et al. discovered that 32% of COVID-19 patients required medical care in the post-acute phase for different diseases. The proportion increased among patients who needed hospitalization for COVID-19 pneumonia. The most frequent sequelae were hypertension, memory difficulty, kidney injury, thrombotic diseases, and cardiac rhythm disorders. Although there were no significant differences between males or females regarding the total incidence of post-acute sequelae, men seem to have a higher risk for respiratory failure and acute kidney injury [42]. 

The data presented by Choudry et al. from their study in the United Kingdom revealed no differences in one-year mortality between COVID-19 patients presenting with STEMI that survived the acute phase compared with COVID-19 negative patients. Their data revealed a 20% rate of in-hospital mortality for COVID patients, and a 4.3% mortality rate in the control group, with a 12% incidence of stent thrombosis for the first group. However, this difference is not sustainable for a long-term outcome. The study also showed higher thrombus burden, multivessel thrombus and in-stent thrombosis—findings sustained in a literature review [16]. Puha et al. [17] found no difference in mortality, cardiac-related readmission, and recurrent coronary events between patients presenting with STEMI in the pandemic era and the control group at one-year follow-up. However, patients presenting STEMI during the COVID-19 pandemic had a higher need for additional PCI during index admission. MACE occurred in 13.4% of the patients from the COVID-19 group assessed at one-year follow-up in this study, and there was a higher readmission rate for heart failure in this group. 

Mortality rates in the general population for AMI patients increased proportionally with the total ischemic time. COVID-19 patients had prolonged first medical contact and door-to-balloon time, probably as a result of fewer patients looking for hospital care and the epidemiological measures required. This may have been one of the reasons for the negative prognosis in patients with COVID-19 and AMI [43]. Patients presenting with AMI with a Killip class greater than II and with COVID-19 disease were more likely to develop a negative prognosis, with higher short-term and long-term mortality. Nanavaty D et al. stated that COVID-19 was independently associated with an increased mortality, longer length of hospitalization and higher total hospitalization cost. In their study, all-cause mortality was significantly higher in the AMI and COVID-19 group, along with higher rates of cardiac arrest, cardiogenic shock, acute organ failure, hemodialysis, and invasive ventilation [44].

Data from the Danish registry reveal that the incidence of acute myocardial infarction 14 days after a positive test for COVID-19 was approximately five times higher than in the pre-COVID-19 era [45]. COVID-19 patients seem to have an increased enzymatic infarct size, assessed by the peak of troponin or creatine kinase levels, lower left ventricular ejection fraction, and higher intracoronary thrombotic burden [46]. 

A more recent trial that compared the efficiency and safety of P2Y12 inhibitors in STEMI patients with COVID-19 disease concluded that the use of Clopidogrel was associated with higher short-term and long-term mortality, with no significant difference in bleeding incidence, compared to other P2Y12 inhibitors. There were no differences in the safety of the P2Y12 inhibitors in the COVID-19-posivite group compared with the -negative group. Although the endothelial dysfunction found in COVID-19-positive patients could lead to an increased risk of major bleeding, in this study, there were no significant differences between COVID-19-positive and -negative patients, regarding bleedings [47].

Regarding readmission rates in patients with ACSs and COVID-19 disease, an 11.4% readmission rate was observed. Out of these, 41% were admissions for cardiac causes, and 59% were for non-cardiac causes. Among the cardiac causes for readmissions within 30 days after index hospitalization, the more frequent were: acute coronary syndromes, arrhythmias and heart failure. Among non-cardiac causes, the most common were infectious diseases, gastrointestinal diseases and respiratory diseases. COVID-19 patients had significantly shorter median times to readmission than non-COVID patients. The presented study conducted by Patel KN observed that the 30-day readmission risk factors were: chronic kidney disease, congestive heart disease, length of index hospitalization ≥ 5 days, and a low median household income [48,49,50,51].

COVID-19 is an important cause of morbidity and mortality, and induces an increase in the incidence of cardiovascular diseases, which is one of the reasons for the need to vaccinate the population on a large scale. One of the most widely studied new topics is the COVID-19 post-immunization side effects. The most frequently described side effects related to COVID-19 vaccines are injection site pain, transient fever, headache, fatigue, lymph node swelling and flu-like symptoms [52]. The most widely discussed, due to their severity, were thrombotic diseases (pulmonary embolism, deep vein thrombosis, venous sinus thrombosis, myocardial infarction) and myopericarditis. The exact causes of myocardial inflammation after vaccination are unclear; it is linked mostly to the molecular resemblance with the spike proteins of SARS-CoV-2 antigens, which trigger an immune response similar to the COVID-19 infection [53]. Post-vaccination myocarditis has an incidence of 12.6 cases per million; it mostly affects younger patients (median age 24) and has a predisposition to affect male patients (79%). Myocarditis was mostly detected as a side effect after the second dose of COVID-19 mRNA vaccines from Pfizer-BioNTech and Moderna [54]. Thrombotic diseases were mostly associated with thrombotic thrombocytopenia observed mostly after AZD1222 and Ad26COV2 vaccines’ administration (Astra Zeneca and Johnson & Johnson) [52,53,54].

## 7. Limitation

This review encountered limitations due to the lack of information on the long-term evolution of patients who presented with acute myocardial infarction and COVID-19 pneumonia. Also, the currently existing studies on this subject have enrolled a relatively small number of patients, and the data from these studies are incomplete. We should mention the need for further research on the importance of the cardiovascular sequelae in the population after SARS-CoV-2 disease.

## 8. Conclusions

The association between COVID-19 disease and acute coronary syndromes has led to higher in-hospital mortality, increased risks of long-term cardiovascular events, and a higher cardiovascular burden. COVID-19 is an independent risk factor for poor prognosis in patients with myocardial infarction. Although some trials have shown no difference in one-year all-cause mortality, this still requires a further study of the long-term cardiovascular complications and prognosis of COVID-19-surviving patients, as well as the relationship between heart disease and this viral infection. 

## Figures and Tables

**Table 2:** Long-term risk of myocardial infarction in COVID-19 survivors.

Authors	1. Garcia S. et al. [8]	2. Xie Y. et al. [9]	3. CinarT. et al. [13]	3. Kiris T. et al. [12]	4. Wang W. et al. [11]
**COVID-19 (+)/COVID-19 (−)**	230/436	153,760/5,637,647	112/609	1686/62	691,455/2,249,533
**Male/Female**	71%/29%	89%/11%	55%/45%	75%/25%	43.2%/56.8%
**MACE (COVID+/COVID−)**	33%/18%	Hazard Ration 1.26 (CI 95%) for non-hospitalized COVID+/2.41 for hospitalized COVID + patient	21.3%/6.3%	22%/22%	Hazard Ratio (CI = 95%) in COVID-19+ was 2.26
**30-day Outcome**	1 out of 3 COVID-19 (+) patients deceased	Incidence of MI increases 3 times for post-COVID-19 patients	Mortality COVID-19 = 21%. Non-COVID-19 = 7.1%	NA	HR for MI at 30-days outcome = 2.32, HR for death at 30 days = 2.067
**Incidence of Miocardial Infarcion in COVID-19 (+) Patient Non-Hospitalized/Hospitalized**	NA	3 times higher MI incidence in hospitalized MI patients	NA	NA	Similar MI incidence for hospitalized/non-hospitalized COVID-19+
**Incidence of Miocardial Infarction at 12-Month Follow-up**	NA	Hazard Ratio (CI 95%) 1.71, burden/1000 pers at 12 M 7.59 for COVID-19+ survivors	NR	6.5% pre-COVID era/5.3% in COVID era	HR (95% CI) = 1.49

**Table 3:** Long-term outcomes of post-COVID-19-positive patients.

Study	1. Xie Y. et al. [9]	2. Wang W. et al. [11]	3. Garcia S et al. [8]	4. Kiris T et al. [12]
Mace	HR (CI 95%) = 1.55 (COVID-19 + 67.67 vs. COVID-19 − 44.19)	10,530 patients HR = 1.871	36%	22%
Cerebrovascular	HR = 1.53 (COVID-19 + 15.95 vs. COVID-19 − 10.48)	4793 patients HR = 1.68	5%	1%
Arhythmyas	HR = 1.69 (COVID-19 + 49.37 vs. COVID-19 − 29.51)	20,927 patients HR = 2.407	NA	NA
Ischemic heart disease	HR = 1.66 (COVID-19 + 18.47 vs. COVID-19 − 11.19)	3651 patients HR = 2.8	6% (Recurrent MI and unplanned revascularization)	15% (Recurrent MI, unplanned revascularization)
Heart failure	HR = 1.72 (COVID-19 + 27.92 vs. COVID-19 − 16.31)	5831 patients HR = 2.29	54%	12%
Thrombotic disease	HR = 2.39 (COVID-19 + 17.07 vs. COVID-19 − 7.19	4599 patients HR = 2.64	NA	NA
Cardiac arrest	HR = 2.45 (COVID-19 + 1.20 vs. COVID-19 − 0.49)	474 patients HR = 1.75	11%	8.50%
Cardiogenic shock	HR = 2.43 (COVID-19 + 0.87 vs. COVID-19 − 0.36)	204 patients HR = 1.98	18%	21%

## Supplementary Materials

The following supporting information can be downloaded at: https://www.mdpi.com/article/10.3390/life14020202/s1, Supplimentary Table S1: PRISMA 2020 CHECKLIST.
